# Predicting *Culex pipiens/restuans* Population Dynamics Using a Weather-Driven Dynamic Compartmental Population Model

**DOI:** 10.3390/insects14030293

**Published:** 2023-03-17

**Authors:** Karin Bakran-Lebl, Lene Jung Kjær, Beate Conrady

**Affiliations:** 1 Institute for Medical Microbiology and Hygiene, AGES—Austrian Agency for Health and Food Safety, 1090 Vienna, Austria; 2 Department of Veterinary and Animal Sciences, University of Copenhagen, Frederiksberg Campus, 1870 Copenhagen, Denmark; 3 Complexity Science Hub Vienna, 1080 Vienna, Austria

**Keywords:** *Cx. pipiens*, *Cx. restuans*, modeling, mosquito, weather, population dynamics, vector

## Abstract

**Simple Summary:**

In this study, we present a compartmental population model for *Cx. pipiens/restuans*, incorporating mosquito life cycle parameters, as well as temperature, precipitation, and geographic latitude. The model was validated against mosquito count data from Cook County (IL, USA). The model fitted the observation data and was able to reproduce between-year differences in the abundance of the *Cx. pipiens/restuans* mosquitoes, as well as the different seasonal trends.

**Abstract:**

Mosquitoes of the genus *Culex* are important vectors of a variety of arthropod-borne viral infections. In most of the northern parts of the USA, *Cx. pipiens/restuans* is the predominant representative of this genus. As vectors, they play a key role in the spreading of arboviruses and thus, knowledge of the population dynamic of mosquitoes is important to understand the disease ecology of these viruses. As poikilotherm animals, the vital rates of mosquitoes are highly dependent on ambient temperature, and also on precipitation. We present a compartmental model for the population dynamics of *Cx. pipiens/restuans*. The model is driven by temperature, precipitation, and daytime length (which can be calculated from the geographic latitude). For model evaluation, we used long-term mosquito capture data, which were averaged from multiple sites in Cook County, Illinois. The model fitted the observation data and was able to reproduce between-year differences in the abundance of the *Cx. pipiens/restuans* mosquitoes, as well as the different seasonal trends. Using this model, we evaluated the effectiveness of targeting different vital rates for mosquito control strategies. The final model is able to reproduce the weekly mean *Cx. pipiens/restuans* abundance for Cook County with a high accuracy, and over a long time period of 20 years.

## 1. Introduction

Mosquitoes of the genus *Culex* (family Culicidae) are important vectors of a variety of infectious diseases, e.g., Rift Valley fever, St. Louis encephalitis, Japanese encephalitis, Western equine encephalitis, and West Nile fever [1,2,3,4]. As vectors, they play a key role in virus transmission cycles, and thus in spreading viral infections. Therefore, in order to understand the epidemiology of mosquito borne arboviruses, knowledge of vector population dynamics is necessary. The main vectors for West Nile virus (WNV) in North America are mosquitoes of the genus *Culex*—especially *Cx. pipiens*, *Cx. tarsalis*, *Cx. quinquefasciatus,* and *Cx. restuans*—but other Culicidae have also been shown to transmit WNV [4,5]. According to their distribution, *Cx. pipiens* is the main vector in the north, *Cx. tarsalis* is the main vector in the west, and *Cx. quinquefasciatus* is the main vector in the south of North America [5,6,7].

Being poikilotherm animals, the population dynamics of *Culex* mosquitoes are highly influenced by ambient temperature [8,9,10]. Laboratory studies have shown the influence of temperature on the development time and survival rates of eggs, larvae, and pupae [11,12,13,14,15]. In adult mosquitoes, temperature affects their lifespan, length of the gonotrophic cycle, and virus transmission rate [11,12,14]. Some *Culex* mosquitoes undergo reproductive diapause, which is also, along with daytime length, affected by temperature [16,17]. Precipitation has also been shown to have a strong impact on *Culex* abundance: a high abundance of *Culex* mosquitoes has been found to be associated with high precipitation occurring several weeks before a capture event [9,10]. The authors of these studies suggested that rainfall increases the water’s surface area and, therefore, possible oviposition sites. Other factors which have been shown to influence the abundance of *Culex* mosquitoes are landscape patterns and land use [9,18,19].

In general, there are two types of models which have been used to describe *Culex* population dynamics: regression models and compartmental models. Regression models have proven to be useful tools in discovering connections between population densities and environmental factors [9,20,21,22]. However, they cannot reveal the biological mechanisms behind those relationships. Regression models are, therefore, of limited use if we want to discover the mechanisms of population dynamics, which is necessary knowledge if we want to manipulate a species population, such as for pest control. Compartmental models, on the other hand, are aimed to represent the mechanisms behind population dynamics. This makes compartmental models ideal for testing as to how the population dynamics of a particular species could be influenced. However, the downside of compartmental models is their need for detailed information on the vital rates of the investigated species. Examples for compartmental *Culex* models are rather rare, requiring detailed information on the location the model is applied to (e.g., the water volume of the breeding site), and/or have only been validated for short time periods [23,24,25]. Therefore, it would be difficult to apply these models to other locations.

In epidemiology, simplified compartmental *Culex* models have been integrated in susceptible, infectious, and/or recovered (SIR) models of mosquito-borne infectious diseases [26,27,28]. In those studies, however, abundance predictions were not compared to actual mosquito capture data, and, thus, these models were not validated. Yu et al. [29] developed a compartmental abundance model for *Cx. pipiens/restuans* that did validate model results against actual mosquito abundance data. Besides mosquito life cycle parameters obtained from field and laboratory studies, this model was, however, solely temperature-driven. The researchers suggested including other factors impacting mosquito abundance, such as the precipitation, landscape, and wind, in order to help improve the predictive power of future models [29].

In this study, we present a compartmental population model for *Cx. pipiens/restuans*, incorporating mosquito life cycle parameters, as well as temperature, precipitation, and geographic latitude. The model was validated against mosquito count data from Cook County (IL, USA). The aim was not to generate a model for a specific area, but rather to produce a model which generates mean values for mosquito abundance that are valid for larger areas (here, we use the county level), which could be used for other areas as well. Thus, we use only easily accessible input parameters (temperature, precipitation, and geographic latitude). This model could therefore be simply integrated in SIR models of *Culex*-borne infectious diseases. Further, we test how manipulating the vital rates influences population size, which is important for mosquito control strategies. Control strategies could be aimed at increasing the mortality rate of certain stages, by using insecticides or biological pest control measurements [30], or, they could be aimed at decreasing the birth rate of mosquitoes, e.g., by releasing sterile individuals (Sterile Insect Technique) [31].

## 2. Materials and Methods

### 2.1. Cx. pipiens/restuans Population Model

To simulate *Cx. pipiens/restuans* population dynamics, we developed a dynamic compartmental population model in R 4.1.2 [32], consisting of a system of ordinary differential equations (OED) with daily time steps. The model is based on the models presented by Laperriere et al. [26] and Rubel et al. [27].

**Figure 1:**
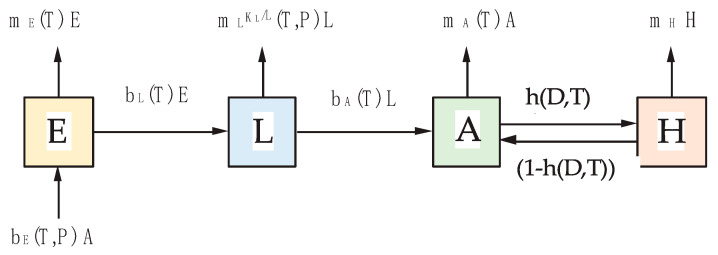
Block diagram of the *Culex* population model, incorporating 4 stages: *E*—eggs, *L*—larvae (inclusive pupal sage), *A*—active adults; *H*—hibernating adults. For definition of the parameters, see Table 1.

Our population model consists of four stage variables: *E*—egg stage, *L*—larval stage, including pupal stage, *A*—active adult mosquitoes, *H*—adult mosquitoes in diapause (Figure 1). Furthermore, we only allowed the proportion of surviving individuals of one stage to have the possibility to change into the next stage.

The daily changes of the number of animals within each stage are described by Equations (1)–(4), where definitions of the parameters used in the equations are described in Table 1.

(1) $$\frac{d\;E}{d\;t}=b_{E}A-m_{E}E-b_{L}E\left(1-m_{E}\right)$$

(2) $$\frac{d\;L}{d\;t}=b_{L}E\left(1-m_{E}\right)-m_{L}{}^{K_{L}/L}L-b_{A}L\left(1-m_{L}{}^{K_{L}/L}\right)$$

(3) $$\frac{d\;A}{d\;t}=b_{A}L\left(1-m_{L}{}^{K_{L}/L}\right)-\left(1-m_{A}\right)hA+\left(1-m_{H}\right)\left(1-h\right)H$$

(4) $$\frac{d\;H}{d\;t}=-m_{H}H+\left(1-m_{A}\right)hA-\left(1-m_{H}\right)\left(1-h\right)H$$

The transition parameters used (Table 1) were estimated from literature values and were influenced only by temperature (*T*), precipitation (*P*), and daytime length (*D*). We combined data from different sources, giving values at different temperatures, and we also used data from other *Culex* species if there was not sufficient data for *Cx. pipiens/restuans* available. By visual inspection of the distribution of the data points, we appraised the form of the function and fitted functions through the data points, using the non-linear least square approach (nls package in R 4.1.2, [32], see Equations (5)–(14)). Estimates were rounded to 2 significant digits. Some functions (e.g., mortality rates) would allow for values above 1 at certain temperatures, which are, from a biological point of view, not possible. Thus, if necessary for a function, it was capped at the value of 1. In this case, we calculated the intersection with a horizontal line at 1. Those interception points represent the lower and upper temperature threshold for the validity of a specific function. Below and above those thresholds, the value was set to 1. We arbitrarily set initial values for *E*, *L*, and *A* to 0.0 and 0.1, for *H* to have a starting point. To minimize the effects of the chosen initial values, we started the model one year before the first mosquito capture data was available, on the date 1 January 1990. For the next sections, the parameters a, b, and c in the function descriptions do not have a biological meaning, but are necessary to shape the parameter functions.

#### 2.1.1. Egg Stage—E

The birth rate of eggs (*bE*) represents the daily egg-laying rate. It can be calculated as the reciprocal value of the length of the gonotrophic cycle. Madder et al. [11], Vinogradova [15] and Elizondo-Quiroga et al. [33] give temperature (*T*) dependent values for the gonotrophic cycle in the *Cx. pipiens* complex, which we used to fit a logistic function:

(5) $$b_{E}\left(T\right)=\frac{a}{1+b\exp\left(c\;T\right)}$$

Various authors have suggested the influence of precipitation on the birth rate [9,10], however, no quantitative data describing this relationship were available. As the availability of suitable *Cx. pipiens/restuans* breeding sites increases with preceding rain events, we included an additional term in order to increase *bE*: the mean sum of precipitation (*P*) of the *k* days before egg laying. Further, to obtain the “actual” birth rate, i.e., the number of female offspring per female per day, we multiplied *bE* by a value equaling half of the mean number of eggs found in an egg raft, as it has been shown that there is an equal number of male and female eggs [34]. For *Cx. pipiens,* this value was estimated to be ≈100 (calculated from Madder et al. [11], Oda and Ueda [35], and Oda et al. [36]).

(6) $$b_{E}\left(T,\;P\right)=100\frac{a+d\;\sum_{i=1}^{k}P_{t-i}}{1+b\exp\left(c\;T\right)}$$

Data on the temperature dependence of the egg mortality rate *(mE)* were given by Madder et al. [11], Shriver and Bickley [37], and Suman et al. [14]. We assumed a polynomic function:

(7) $$m_{E}\left(T\right)=\left\{\begin{array}{c} a+bT+cT^{2},\;\mathrm{if}\;tr_{l\left(m_{E}\right)}<T<tr_{u\left(m_{E}\right)}\\ 1,\;\mathrm{otherwise} \end{array}\right.$$

where *tr_l_* and *tr_u_* are lower and upper temperature thresholds for the validity of the function, respectively, as the mortality rate cannot exceed 1.

#### 2.1.2. Larval Stage—L

In our model, we combined the larval and pupal stages of the mosquitos into one compartment (hereafter only referred to as the “larval stage”). The larval birth rate (*bL*) is the daily hatching rate of eggs into larvae, and is calculated as the reciprocal value of the duration of the egg stage. The temperature-dependent larval birth rate has been well documented, and can be described by a logistic function [11,14,15,37,38,39,40]:

(8) $$b_{L}\left(T\right)=\frac{1}{1+a\exp\left(b\;T\right)}$$

The temperature-dependent mortality rate of the larval stage (*mL*) has been described for *Cx. pipiens* by Madder et al. [11], Olejniček and Gelbič [41], and Tamarina [42]). The data fitted a rational function:

(9) $$m_{L}\left(T\right)=\left\{\begin{array}{c} \frac{a+b\;T}{\left(T-T_{min.larv}\right)\left(T-T_{max.larv}\right)},\;\mathrm{if}\;tr_{l\left(m_{L}\right)}<T<tr_{u\left(m_{L}\right)}\\ 1,\;\mathrm{otherwise} \end{array}\right.$$

where *tr_l_* and *tr_u_* are lower and upper temperature thresholds for the validity of the function, respectively, as the mortality rate cannot exceed 1.

This function required information on the minimum and maximum temperature for larval survival (T_min.larv_ and T_max.larv_). Mean values found in the literature were T_min.larv_ = 8.7 °C [11,43,44,45] and T_max.larv_ = 36.4 °C [43,46]. Mortality rates are likely to be biased and highly underestimated under lab conditions. For *Cx. tarsalis,* it has been shown that only 10% of larval mortality in the field is due to abiotic factors [34]. Thus, we increased obtained mortality rates under lab conditions for larvae by a factor of 10.

The mortality of larvae has been shown to increase with larval density [11,47,48], which we incorporated in our model as well. Suitable densities for larval development are likely to depend on habitat structure, and, thus, the larval carrying capacity *KL*. Unfortunately, this parameter, as well as its relation to the number of adult mosquitoes, is unknown. Assuming a correlation between the number of larval and adult mosquitoes, and, thus, between the carrying capacity of the two stages, we estimated a scaling factor, which gives *KL* in relation to the capture rate of adult females. Using a scaling factor instead of estimating *KL* directly makes the results of this study applicable to other areas. We used the mean number of captured *Cx. pipiens/restuans* during their peak abundance (i.e., in July), which was found to be 5.75 individuals per day for our study area. As the volume of larval habitat increases with preceding rain events, we included an additional term to increase *KL:* the mean sum of precipitation of the k preceding days. Please note that *k* is the same as for Equation (6).

(10) $$K_{L}\left(P;t\right)=a\;5.75+b\;\frac{1}{k}\sum_{i=1}^{k}P_{t-i}$$

#### 2.1.3. Adult Stages—A and H

The birth rate of adult mosquitoes *(bA)* represents the hatching rate of mosquitoes, from larvae to imagoes. Analogous to the larval birth rate, the adult birth rate can be calculated as the reciprocal value of the duration of the larval period. Temperature-dependent durations of the larval period for the *Cx. pipiens* complex are given in [13,38,40,46,49]. Again, the data fitted a logistic function:

(11) $$b_{A}\left(T\right)=\frac{1}{1+a\exp\left(b\;T\right)}$$

Almir’on and Brewer [38], Gunay et al. [50], Oda et al. [51], Reisen et al. [12] and Suman et al. [14] give data on the temperature dependence of adult mortality rates *(mA)*. We assumed a polynomic function:

(12) $$m_{A}\left(T\right)=\left\{\begin{array}{c} a+bT+cT^{2},\;\mathrm{if}\;tr_{l\left(m_{A}\right)}<T<tr_{u\left(m_{A}\right)}\\ 1,\;\mathrm{otherwise} \end{array}\right.$$

where *tr_l_* and *tr_u_* are lower and upper temperature thresholds for the validity of the function, respectively, as the mortality rate cannot exceed 1.

In winter, *Cx. pipiens* and *Cx. restuans* undergo reproductive diapause [16,17,48]. This diapause is mainly characterized by the arrest of ovarian development, but also accompanied by other metabolic and behavioral changes [15]. Ovarian development, which we will use as a proxy for the occurrence of diapause, is dependent on the temperature (*T*) and daytime length (*D*), as investigated for *Cx. pipiens* by Eldridge [52], and a logistic function can be fitted to the data:

(13) $$\;h\left(T,D\right)=\frac{1}{1+a\exp\left(b\;T+c\;D\right)}$$

Therefore, *h* is the rate at which an adult mosquito changes from the active stage, *A,* into the diapausing (hibernating) stage, *H*, and 1 h is the rate at which adult mosquitoes terminate diapause. The mosquitoes spend hibernation in natural and artificial shelters, where they are protected from the harsh climatic conditions during winter [15]. Bailey et al. [53] reported that for diapausing *Cx. pipiens,* the proportions of individuals surviving the winter (14 December—8 March; equates to 84 days) were 0.157, 0.224 and 0.247. Thus, we can calculate the daily survival rate during diapause in winter, and hence the daily winter mortality rate:

(14) $$m_{H}=1-exp\left(\frac{\log\left(a\right)}{84}\right)$$

### 2.2. Model Evaluation

#### 2.2.1. Mosquito Count Data

For the evaluation of our model, we used mosquito count data from Cook County (IL, USA), provided by the Desplaines Valley Mosquito Abatement District (P. Geery, 2011, pers. comm.). This agency had long-term capture data available, as it has been capturing mosquitoes with New Jersey light traps at 25 trap sites since 1980.

However, not all trap sites were continuously operated during all years, and, thus, only those trap sites providing continuous time series for at least 20 years were selected, resulting in six capture sites (details on the trap location, data preparation and mosquito data selection are published by Lebl et al. [22]). For this study, the numbers of female mosquitoes were averaged over these six capture sites. Although the sites are not uniformly distributed within the county, the resulting average time series was considered to be representative for Cook County. Of the 81,022 *Culex* females caught at the trap sites between 1991 and 2010, 97% were *Cx. pipiens/restuans*, and the rest were *Cx. territans*, *Cx. tarsalis*, *Cx. erraticus,* and *Cx. salinarius*. The catches of the morphologically rather similar species *Cx. pipiens* and *Cx. restuans* were combined, as distinguishing them based on morphological characteristics is rather imprecise.

#### 2.2.2. Environmental Data

The mean daily temperature (*T*) and daily sum of precipitation (*P*) data were taken from the weather station of the Chicago O’Hare International Airport (WMO No. 72530 [54]; 87.900° W, 41.983° N and altitude 205 m). The daytime length, *D* (in hours), is a function of the sun’s declination, *E,* and the geographic latitude ϕ, and was calculated using the CBM model described by Forsythe et al. [55]:

(15) $$D=24-\frac{24}{\pi }\mathrm{acos}\left(\frac{\sin\left(0.8333\right)+\sin\left(\varphi \right)\sin\left(\epsilon \right)}{\cos\left(\varphi \right)\cos\left(\epsilon \right)}\right)$$

with the declination of the sun ε calculated as a function of the calendar day *d*:

(16) $$\epsilon =\mathrm{asin}\left(0.39795\;\cos\left(0.2163108+2\;\mathrm{atan}\left(0.9671396\;\tan\left(0.00860\;\left(d-186\right)\right)\right)\right)\right)\;$$

#### 2.2.3. Model Calibration and Validation

We used function estimates as initial values for our model. For the parameter associated with precipitation, our initial value was chosen as 0, i.e., precipitation had no effect. The model results had a temporal resolution of 1 day. Due to the high inter-day variation in the capture rates [22], we calculated the weekly mean of the capture rates and model estimates to be used for further analysis. To evaluate model fit, we calculated the root mean square error (*RMSE*) between the mosquito capture data and the model estimates for the active adult mosquitoes, A, as well as the Spearman’s correlation coefficient, *rS* (as the count data followed a non-Gaussian distribution).

The individual function parameters (a, b, and c) for the transition parameters were estimated based on literature values obtained at laboratory conditions, thus, it is possible that the parameters of these functions would be different at field conditions. Consequently, we allowed for the function parameters to vary within the standard error (SE) of their estimated value (Table 2). To divide the data set into a training and a test set, we distinguished between even and odd years. In a previous study, we used a random process to choose odd years as the test data, and we retained this division in order to make the results comparable [22].

With the training data set, a genetic algorithm was used, in order to identify the parameter combination that fitted the data best (i.e., minimizing *RMSE*). This algorithm is a search heuristic inspired by natural evolution that is used for optimization problems [56] where a given number of possible solutions (populations) to a given problem exists. The values generating the best solutions for the optimization problem (this study: lowest *RMSE*) undergo “mutations” and “recombinations” to produce a new population to be tested. This procedure is continued for a chosen number of iterations. For the calculation of the genetic algorithm, we used program R 4.1.2 [32], with the package genalg [57].

The genetic algorithm was run 30 times, each with a population size of 500 and 2000 iterations. Validation of the stability of the *RMSE* were performed depending on the number of iterations and runs (see Supplementary Material Figure S1). The model calibration with the lowest *RMSE* was chosen as the final model, and the model’s fit was assessed using the test data. To compare variations in estimates between the 30 runs, we calculated the relative standard deviation (*RSD*).

#### 2.2.4. Estimating the Effect of Mosquito Control Strategies

Mosquito control strategies could either be aimed to reduce the birth rate (*bE*) or increase the mortality of the different stages (*mE*, *mL*, *mA*, *mH*). After calibrating the vital rate functions, we decreased the birth rate and increased the mortality rates by 5%, 10%, 25%, 50%, and 75%, both individually and with some of these changes combined. Mortality rates greater than one were set to one. To test the effect of the modified vital rates on the population size, we compared the total number of adult mosquitoes (*A*) during the study period 1991–2010, as predicted by our model, with the models in which the altered rates were used. The total number of mosquitoes in the model after calibration was set to be 100%, and the population size obtained with the altered vital rates was calculated as a proportion of this size.

## 3. Results

The initial function parameter estimates using non-linear least-squares can be seen in Table 2. Using these initial function parameters, without any calibration, produced a seasonal pattern of mosquito abundance, which fitted the capture data passably. We found (with the initial estimates) an *RMSE* = 5.1814 and an *rS* = 0.832 for the training data, and an *RMSE* = 5.1840 and an *rS* = 0.826 for the test data.

**Figure 2:**
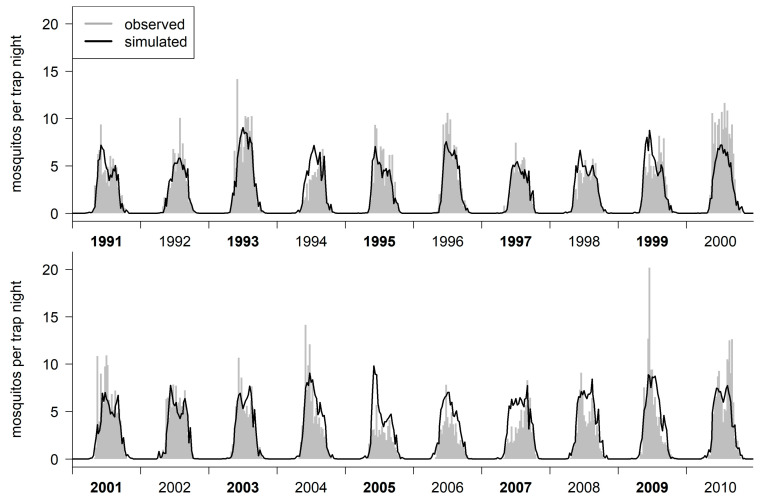
Standardized weekly averaged observation data and final model results. Years written in regular type indicate the years for the training data set; years in bold type indicate the test data set.

With parameter calibration (parameters are shown in Table 2) the estimated abundance of active mosquitoes (*A*), as calculated by the final model, fitted the observations from the training data well (*RMSE* = 1.3457, *rS* = 0.905; Figure 2).

The results from the test data set showed an equally good fit (*RMSE* = 1.437, *rS* = 0.900). For the whole observation period (1991–2010, training and test data combined), we calculated the *RSME* = 1.393 and *rS* = 0.902.

This final model was able to reproduce inter-year variability in terms of the abundance of the *Cx. pipiens/restuans* mosquitoes, as well as in terms of the different yearly trends. However, it overestimated mosquito abundance in years with very low capture rates, and, thus, with potentially low abundance (1994, 2005, 2007), while underestimating the abundance for some years with very high mosquito capture rates (2000, 2009).

**Figure 3:**
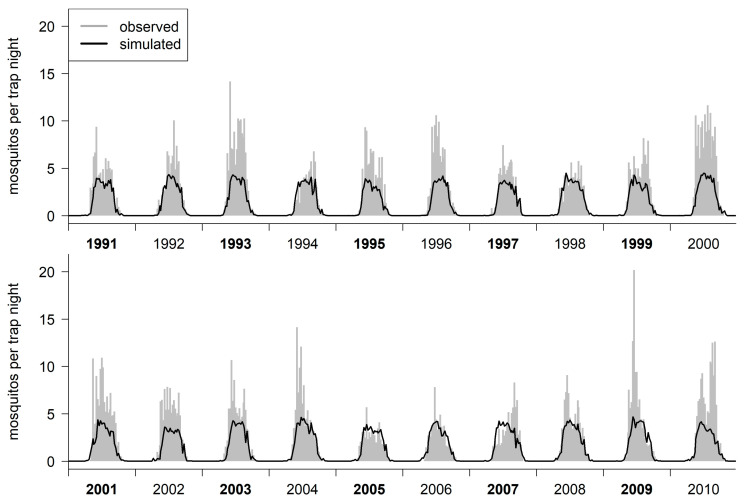
The estimated abundances of active mosquitoes, using the capture data from the test data set, without including precipitation.

To investigate the importance of including precipitation into the model, we set the parameters associated with precipitation to zero (*KL*(*b*) and *bE*(*d*)), while using all other parameters from the final model. This model without precipitation still resulted in a high correlation with the capture data from the test data set (*rS* = 0.899), but with a lower *RMSE* = 1.898 (Figure 3).

The estimates for the calibrated model parameters, from the 30 runs conducted using the genetic algorithm, were mostly located within a small section of their possible range (Table 2). The highest relative variation was found for parameter *a* of the function for *h*, which modulates the horizontal position of the inflection point. Parameters *a* and *b* of the *KL* function, which we had to choose to our best knowledge, also showed a higher variation. Surprisingly, parameter *k*, which is part of the *bE* and *KL* function and gives the sum of the days with precipitation, showed only a small variation, despite the large possible range we allowed for with this parameter, due to missing preliminary information.

**Figure 4:**
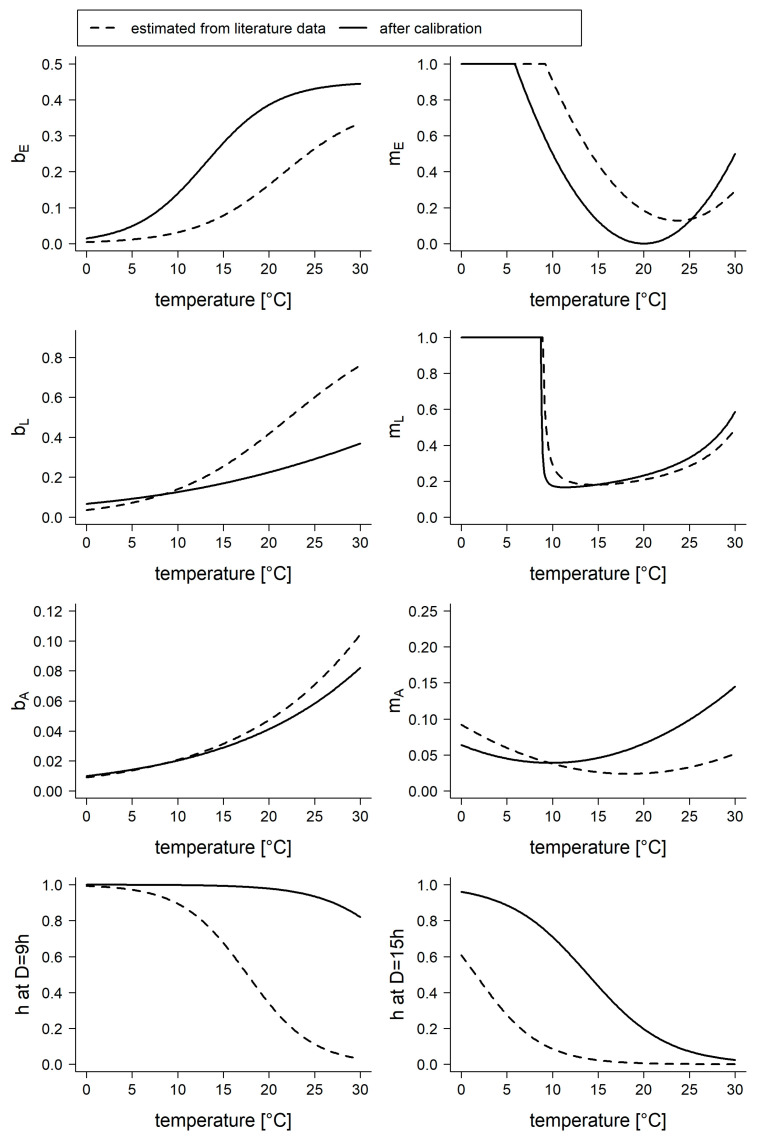
Functions used to describe the transition parameters (see Table 1). Dashed lines represent the estimated functions fitted to literature values (*mL* multiplied by a factor of 10), and solid lines represent the functions after calibration with the training data set.

The resulting functions for the birth rates used in the final model showed that temperature developmental time for adults (*bA*) showed little deviation from the literature (Figure 4), whereas temperature developmental time was lower for larvae (*bL*) and higher for eggs (*bE*) compared to the initial functions obtained from literature. For the mortality rates, the estimated function parameters for *mL* were nearly identical to the non-calibrated function parameters, while the mortality rates for the eggs were lower, allowing the eggs to survive at temperatures below 10°; therefore, the optimal temperature for egg survival was shifted to lower temperatures.

The highest modification was found for *h*, the transition rate to and from the diapausing stage. In the final model, the function for *h* implied that under short day conditions, most mosquitoes were in the diapausing stage, which was nearly independent from the temperature. At long daytime lengths, on the other hand, temperature had a high impact on the transition between the stages *A* and *H*.

When compared to values from literature, the function of the diapausing rate (*h*) was skewed to the right, suggesting that, even at higher temperatures, some mosquitoes are inactive. To reduce the population of *Cx. pipiens/restuans*, the vital rates would have to be influenced considerably (Table 3). The most effective modification is a simultaneous increase of *mL* and *mA*, followed by other combinations, including an increase of *mL*. The model simulation showed that the population size is rather robust against decreasing *bE* or an increasing *mE*.

## 4. Discussion

The dynamic compartmental model presented in this study is able to reproduce the weekly mean *Cx. pipiens/restuans* abundance for Cook County at a high accuracy and over a long time period of 20 years. It only needs a few, easily accessible input parameters, namely, temperature, precipitation, and daytime length (as a function of the geographic latitude). Due to the averaging of values over several trap locations, we were able to build a model that was independent from trap-site-specific effects on mosquito abundance, such as small water bodies. Therefore, our model can be an important tool to predict *Cx. pipiens/restuans* abundance, and, thus, the spread of diseases transmitted by this vector species. In a previous study, we used lagged weather data and a Poisson regression model to generate a predictive model for *Cx. pipiens/restuans* abundance [22]. The performance of the Poisson model is directly comparable to the compartmental model presented in this study, as the same mosquito capture data were used to generate and to evaluate the models. The fit of both model types is similar (the Poisson model had a slightly poorer performance, with an *RMSE* = 1.752 for the test data set), but the Poisson model needed two additional input parameters, namely, relative humidity and wind speed. A further benefit is that this compartmental model can easily be included in SIR models for pathogens transmitted by *Cx. pipiens/restuans*.

The final model in this study showed that the highest difference in vital rates from literature values was found for the transition between the active and diapausing adult stage. This was not surprising, as we had only scarce data available to generate our initial function, which also did not give the proportion of diapausing females per se, but instead gave proportion of the females with arrested ovarian development. Further, studies conducted under laboratory conditions usually work with constant parameters (e.g., constant temperature, light or dark), while field conditions are characterized by changing conditions, such as temperature variations during the day or dim light conditions at dawn and dusk. This variation in the conditions is likely to modify the temperature and photoperiod dependence observed in the laboratory. This does not only apply for the diapausing rate, but likely for all included vital rates [58], and could explain other, not so strongly pronounced, deviations in the vital rates when compared to literature values.

Including precipitation in our model (for estimating the egg-laying rate *bE* and the carrying capacity *KL*) considerably contributed to the fit of the final model to the observed mosquito abundances. Interestingly, although our settings would have allowed us to include accumulated rainfall from up to 20 days prior, only rainfall during the previous day was selected through the genetic algorithm. Both *Cx. pipiens* and *Cx. restuans* use a wide variety of water bodies as breeding sites, ranging from larger, semi-permanent pools to small (artificial) containers, such as tin cans [59]. As small, temporal water bodies are most affected by the most recent rainfall events, our study emphasizes the importance of those temporal water bodies as breeding sites for *Cx. pipiens/restuans*. As with the other environmental factors, the variation in the distribution of rainfall likely modulates the effect of the total sum of precipitation during a certain period. For example, as shown by Valdez et al. [60], a high variability increases the production of mosquitoes at a low rainfall regime, while the opposite behavior takes place in the case of high precipitation.

Importantly, the investigated mosquito population has been subject to mosquito control measures. However, during the whole study period, the control methods have been constantly changing, and so have their efficiency. Control measurements have been mainly targeting the larval stage of mosquito development, but since 2002, there has been also occasional spraying of an adulticide (P. Geery, pers. comm.). Aberrations in our model’s results from the observation data could partly be caused by yearly differences in the use or efficiency of the control strategies. We also need to emphasize that our model represents a “managed” *Cx. pipiens/restuans* population. This needs to be taken into account when applying the model to other areas and regions. However, in many areas (at least in the USA) where mosquito-borne diseases such as WNV occur, there is some kind of mosquito control, thus, the model might be highly applicable for such areas. By manipulating the vital rates in our model, we could show that additional control measures could result in a further reduction in mosquito abundance when compared to the use of the control measures that have been already applied in Cook County during 1991–2010. As our results showed, the resulting effects on the population size are nearly linear, thus, our results would apply to unmanaged *Cx. pipiens/restuans* populations as well. Mosquito management strategies typically consist of a combination of applying larvicide and adulticide, as well as efforts to reduce breeding sites, but the efficiency of these methods varies [30,61]. The results of our study suggest that focus should be on increasing the mortality rates of larvae and adults. However, to obtain a sustainable effect, the mortality rates would have to be strongly increased.

The *Cx. pipiens/restuans* abundance model by Yu et al. [29] resembles the model created in this study, however, it did not include precipitation, which in our model increased model fit. As with our model, their abundance model showed some restraints regarding predictive power in some years, but no *RMSE* or correlation coefficients were given, so we cannot directly compare our model to their work. However, as Yu et al. [29] suggest, discrepancies in observed and estimated data could be due to skewness in the surveillance data, caused by some of the traps capturing a disproportionately large number of mosquitoes relative to other traps included in the surveillance program. In our study, we aimed to reduce the bias caused by inhomogeneous traps, by altering the selection process of the data used [22], however, we cannot avoid this bias completely. Additionally, poor predictive power in our model for some years could be due to differences in mosquito control measures as described above, or a combination of unusual weather phenomena, resulting in unusually low or high mosquito numbers. Whereas control measures can be accounted for in models, given detailed information, unusual weather phenomena can be very hard to predict and capture in a model.

Studies evaluating mosquito population models with capturing face the problem that the capture data does not represent the whole (female) mosquito population size, as mosquito traps usually target host-seeking females. Further, trap types differ in their efficiency, which has to be considered when comparing model output with capture data obtained from other trap types. A further challenge will be to test this model in different areas and regions [62,63,64]. As the geographic latitude is already included in this model, it should be rather straight forward to apply this model to other areas. Further, we intend to adapt this model for other *Culex* species, especially for the other important WNV vectors, *Cx. tarsalis* in the west and *Cx. quinquefasciatus* in the south of the United States. Further development of this model by implementing the effect of varying environmental conditions would likely increase the predictive power.

## 5. Conclusions

The presented dynamic compartmental model was able to reproduce the weekly mean *Cx. pipiens/restuans* abundance for Cook County at high precision. As it only needs a few input parameters, namely, temperature, precipitation, and latitude, it can be adapted to other regions and easily implemented into SIR models for pathogens transmitted by *Cx. pipiens/restuans* mosquitoes.

## Figures and Tables

**Table 1:** Definition of the transition parameters used in the *Culex* population model. Model parameters are per capita rates in units days^−1^.

Parameter ^1^	Description
bE (T, P)	birth rate eggs—reciprocal value of the length of the gonotrophic cycle multiplied with the number of female eggs laid per egg raft
mE (T)	mortality rate of eggs
bL (T)	birth rate larvae—reciprocal value of the length of the egg stag
mL (T, KL)	mortality rate of larvae and pupae combined
KL (P)	carrying capacity of the larvae
bA (T)	birth rate adults—reciprocal value of the length of the larval and pupal stage combined
mA (T)	mortality rate of active adult mosquitoes
h (T, D)	diapausing rate—rate of adult mosquito leaving the active stage and entering the diapause stage
mH	mortality rate of adult mosquitoes during winter diapause

^1^ Information on parameters, depending on environmental factors, is given in parentheses: T—daily mean ambient temperature (°C), P—daily sum of precipitation (mm) and D—daytime length (h).

**Table 2:** Parameter estimates for Equations (6)–(14) for the *Cx. pipiens*/*restuans* mosquito model. The estimates are the values for a specific function fitted to literature data, and the according lower and upper margin (estimate ± SE). Values estimated to our best knowledge, i.e., not based on literature data, are indicated by an asterisk (*). Furthermore, the model calibration with the lowest *RMSE* (in bold), as well as the minimum, maximum, mean, standard deviation (*SD*) and relative standard deviation (*RSD*), obtained by the 30 runs of the genetic algorithm (training data set), are shown.

Func.	Par.	Estimate	Lower	Upper	Calib	Min	Max	Mean	SD	RSD
*b_E_*	a	3.9 × 10^−1^	3.4 × 10−1	4.5 × 10^−1^	**4.5 × 10^−1^**	4.2 × 10^−1^	4.5 × 10^−1^	4.4 × 10^−1^	7.9 × 10^−3^	1.8 × 10^−2^
	b	9.5 × 10^1^	2.9 × 10^1^	1.6 × 10^2^	**3.0 × 10^1^**	2.9 × 10^1^	3.2 × 10^1^	3.0 × 10^1^	8.6 × 10^−1^	2.9 × 10^−2^
	c	−2.1 × 10^−1^	−2.6 × 10^−1^	−1.7 × 10^−1^	**−2.6 × 10^−1^**	−2.6 × 10^−1^	−2.4 × 10^−1^	−2.5 × 10^−1^	5.3 × 10^−3^	−2.1 × 10^−2^
	d *	0.0 × 10^0^	0.0 × 10^0^	1.0 × 10^−1^	**1.0 × 10^−1^**	5.0 × 10^−2^	1.0 × 10^−1^	9.7 × 10^−2^	9.1 × 10^−3^	9.4 × 10^−2^
*b_E_, K_L_*	k *	0.0 × 10^0^	0.0 × 10^0^	2.0 × 10^1^	**1.1 × 10^0^**	8.4 × 10^−1^	1.8 × 10^0^	1.2 × 10^0^	2.5 × 10^−1^	2.1 × 10^−1^
*m_E_*	a	2.5 × 10^0^	2.0 × 10^0^	2.9 × 10^0^	**2.0 × 10^0^**	2.0 × 10^0^	2.5 × 10^0^	2.1 × 10^0^	1.1 × 10^−1^	5.4 × 10^−2^
	b	−2.0 × 10^−1^	−2.4 × 10^−1^	−1.6 × 10^−1^	**−2.0 × 10^−1^**	−2.2 × 10^−1^	−1.8 × 10^−1^	−2.0 × 10^−1^	1.1 × 10^−2^	−5.6 × 10^−2^
	c	4.2 × 10^−3^	3.3 × 10^−3^	5.0 × 10^−3^	**5.0 × 10^−3^**	4.1 × 10^−3^	5.0 × 10^−3^	4.9 × 10^−3^	2.4 × 10^−4^	5.0 × 10^−2^
*b_L_*	a	2.7 × 10^1^	1.4 × 10^1^	4.0 × 10^1^	**1.4 × 10^1^**	1.3 × 10^1^	3.3 × 10^1^	1.6 × 10^1^	4.2 × 10^0^	2.7 × 10^−1^
	b	−1.5 × 10^−1^	−1.7 × 10^−1^	−1.3 × 10^−1^	**−7.0 × 10^−2^**	−1.7 × 10^−1^	−5.9 × 10^−2^	−1.2 × 10^−1^	3.2 × 10^−2^	−2.7 × 10^−1^
*m_L_*	a	1.9 × 10^1^	−3.6 × 10^0^	4.1 × 10^1^	**3.1 × 10^1^**	2.5 × 10^1^	3.3 × 10^1^	2.8 × 10^1^	2.3 × 10^0^	8.1 × 10^−2^
	b	−2.9 × 10^0^	−3.8 × 10^0^	−2.0 × 10^0^	**−3.7 × 10^0^**	−3.8 × 10^0^	−3.7 × 10^0^	−3.7 × 10^0^	4.1 × 10^−2^	−1.1 × 10^−2^
*K_L_*	a *	1.0 × 10^0^	1.0 × 10^0^	5.0 × 10^1^	**1.0 × 10^0^**	1.0 × 10^0^	1.8 × 10^0^	1.2 × 10^0^	2.2 × 10^−1^	1.8 × 10^−1^
	b *	1.0 × 10^0^	1.0 × 10^0^	7.0 × 10^1^	**3.6 × 10^1^**	3.6 × 10^1^	3.7 × 10^1^	3.6 × 10^1^	5.7 × 10^−1^	1.6 × 10^−2^
*b_A_*	a	1.1 × 10^2^	7.3 × 10^1^	1.5 × 10^2^	**1.0 × 10^2^**	7.5 × 10^1^	1.5 × 10^2^	1.1 × 10^2^	2.2 × 10^1^	2.1 × 10^−1^
	b	−8.5 × 10^−2^	−9.8 × 10^−2^	−7.3 × 10^−2^	**−7.3 × 10^−2^**	−7.4 × 10^−2^	−7.3 × 10^−2^	−7.3 × 10^−2^	3.9 × 10^−4^	−5.2 × 10^−3^
*m_A_*	a	9.2 × 10^−2^	6.4 × 10^−2^	1.2 × 10^−1^	**6.4 × 10^−2^**	6.4 × 10^−2^	8.5 × 10^−2^	6.8 × 10^−2^	5.0 × 10^−3^	7.3 × 10^−2^
	b	−7.5 × 10^−3^	−1.0 × 10^−2^	−5.0 × 10^−3^	**−5.1 × 10^−3^**	−5.3 × 10^−3^	−4.9 × 10^−3^	−5.0 × 10^−3^	1.0 × 10^−4^	−2.1 × 10^−2^
	c	2.0 × 10^−4^	1.5 × 10^−4^	2.6 × 10^−4^	**2.6 × 10^−4^**	2.6 × 10^−4^	2.6 × 10^−4^	2.6 × 10^−4^	8.7 × 10^−7^	3.4 × 10^−3^
*m_H_*	a	1.8 × 10^−2^	1.7 × 10^−2^	2.0 × 10^−2^	**1.7 × 10^−2^**	1.7 × 10^−2^	2.0 × 10^−2^	1.8 × 10^−2^	8.1 × 10^−4^	4.5 × 10^−2^
*h*	a	8.4 × 10^−6^	−6.7 × 10^−6^	2.4 × 10^−5^	**8.8 × 10^−8^**	8.0 × 10^−8^	3.2 × 10^−7^	1.2 × 10^−7^	5.6 × 10^−8^	4.6 × 10^−1^
*h*	b	2.8 × 10^−1^	2.3 × 10^−1^	3.3 × 10^−1^	**2.3 × 10^−1^**	2.3 × 10^−1^	2.4 × 10^−1^	2.4 × 10^−1^	1.9 × 10^−3^	8.0 × 10^−3^
*h*	c	7.5 × 10^−1^	6.2 × 10^−1^	8.7 × 10^−1^	**8.7 × 10^−1^**	7.8 × 10^−1^	8.7 × 10^−1^	8.5 × 10^−1^	2.4 × 10^−2^	2.9 × 10^−2^

**Table 3:** Effect of mosquito control strategies, increasing/decreasing different vital rates by a certain percentage, on the relative population size. The percentage of active mosquitoes during the study period (1991–2010) was obtained by using the altered rate in relation to the number of mosquitoes predicted by the model after calibration.

	5%	10%	25%	50%	75%
decrease *b_E_*	99.6	99.2	97.8	94.2	86.5
increase *m_E_*	99.7	99.5	98.6	97.4	96.3
increase *m_L_*	96.2	92.6	82.6	68.4	56.5
increase *m_A_*	96.2	92.7	83.6	71.9	63.1
increase *m_H_*	99.1	98.3	95.9	92.3	89.1
decrease *b_E_* + increase *m_E_*	99.3	98.7	96.4	91.4	82.2
decrease *b_E_* + increase *m_L_*	95.8	91.8	80.7	64.2	48.0
decrease *b_E_* + increase *m_A_*	95.8	92.0	81.6	67.0	52.6
decrease *b_E_* + increase *m_H_*	98.7	97.5	93.6	85.9	73.8
increase *m_E_* + increase *m_L_*	95.9	92.1	81.5	66.5	54.2
increase *m_E_* + increase *m_A_*	95.9	92.2	82.4	69.8	60.4
increase *m_E_* + increase *m_H_*	98.8	97.7	94.6	89.8	85.6
increase *m_L_* + increase *m_A_*	92.6	85.8	69.1	49.3	35.8
increase *m_L_* + increase *m_H_*	95.3	91.0	79.1	62.9	49.7
increase *m_A_* + increase *m_H_*	95.4	91.1	80.4	66.8	56.8

## Data Availability

The data presented in this study are available on reasonable request from the corresponding author.

## Supplementary Materials

The following supporting information can be downloaded at: https://www.mdpi.com/article/10.3390/insects14030293/s1, Figure S1: Validation of the stability of root-mean-square error (*RMSE*) depending on the number of iterations and runs.
