# Supplementary material for: Predicting Culex pipiens/restuans Population Dynamics Using a Weather-Driven Dynamic Compartmental Population Model
Source: Insects. 2023 Mar 17;14(3):293. doi: 10.3390/insects14030293 (PMC10056620; doi:10.3390/insects14030293)

Supplementary Information

Figure S1: Validation of the stability of root-mean-square error (RMSE) depending on the number of iterations and runs

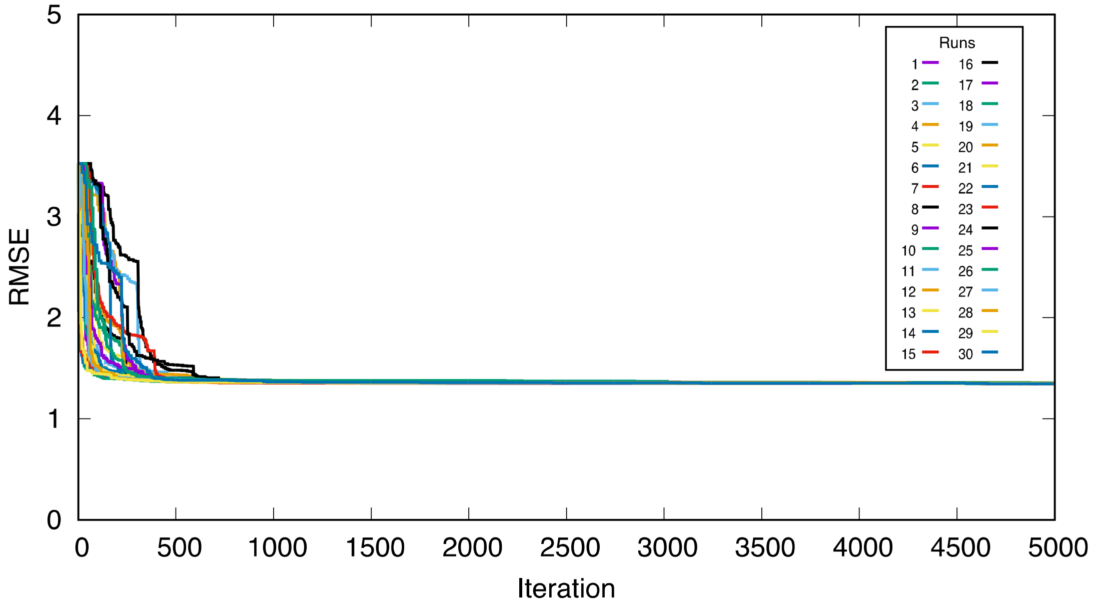

Supplement: Supplementary file 1 [file insects-14-00293-s001.zip › insects-2283732-supplementary.pdf]
